# “4 × 4 vasovasostomy”: A simplified technique for vasectomy reversal

**DOI:** 10.4103/0970-1591.70564

**Published:** 2010

**Authors:** Rajeev Kumar, Satyadip Mukherjee

**Affiliations:** Department of Urology, All India Institute of Medical Sciences, New Delhi, India

**Keywords:** Azoospermia, contraception, male infertility, vasectomy

## Abstract

**Objectives:**

An ideal vasovasostomy procedure requires precise mucosal approximation with a watertight anastomosis. The standard two-layer microdot technique requires multiple sutures within each layer and is a technically difficult operation to perform. We describe a two-layered technique which adheres to the principles of tubular anastomosis, but is simpler and provides excellent results.

**Materials and Methods:**

Patients with secondary infertility following a previous vasectomy underwent the modified two-layered vasovasostomy. Two 8-0 polyamide sutures were placed at 5 and 7 o’clock positions in the sero-muscular layer to approximate the two ends of the vas. Next, four double-armed, 10-0 polyamide sutures were sequentially placed, inside out in the mucosa of the vasal ends, at 3, 6, 9, and 12 o’clock positions and tied. Two additional sero-muscular sutures were placed at 1 and 11 o’clock positions to complete the anastomosis. Patients with a suspected proximal block in the epididymis underwent a vasoepididymostomy. Semen analysis was performed at 6 weeks after surgery.

**Results:**

Between the period February 2008 and August 2009, eight men underwent vasectomy reversal using the 4 × 4 technique. The procedure was performed bilaterally in six men whereas two patients underwent a two-suture, longitudinal intussusception vasoepididymostomy on the second side. Mean operative time was 90 min per patient. All men had sperm in the ejaculate at the first semen analysis. There were no complications.

**Conclusions:**

The “4 × 4” modified two-layer vasovasostomy is a simple technique that can be performed in quick time with excellent results. It may allow a common ground between the complex microdot two-layer technique and the over-simplified single-layer procedure.

## INTRODUCTION

Vasectomy is a relatively uncommon technique of contraception in India.[1] A number of surveys have found this to be the choice of under 5% of all couples using contraception in developing countries.[2,3] Increasing literacy, acceptance of the role of the male partner in contraception, and availability of the no-scalpel technique for vasectomy are some of the reasons for a potential increase in its utilization.

One of the advantages of vasectomy as a “permanent” method of contraception is also the relative ease of its reversibility. Vasectomy reversal surgeries have usually been reported to have greater than 90% patency rates.[4–7] This ability to restore fertility can potentially be an important advantage in promoting its use as a contraceptive method.

Vasectomy reversal requires a precise anastomosis between the proximal and distal ends of the vas deferens. The discrepancy in the lumen of the normal distal and dilated proximal ends often makes this difficult. One of the most well-known techniques for this surgery is the multilayer microdot procedure described by Goldstein *et al*.[8] While the authors did achieve a success rate of 99.5%, this technique required the placement of 22–24 microsutures around the vas and was beyond the technical capability of most surgeons. A number of techniques have been subsequently described in an attempt to simplify this procedure. We perform vasovasostomy using a simple two-layer, 8 microsuture technique. In this study, we describe our technique and present the results.

## MATERIALS AND METHODS

Patients presenting to us with secondary infertility following a previous vasectomy were evaluated for suitability for surgical reversal. The duration of vasectomy was recorded, and a physical examination was performed to locate the site of vasectomy and confirm the presence of vas deferens above it. A semen analysis was performed in all patients, and a follicle stimulating hormone level was obtained if the testis felt flabby. An informed written consent was obtained from all patients before surgery.

Vasectomy reversal was performed under general anesthesia on a day-care basis. A vertical incision was made at the base of the scrotum, away from the midline to deliver the testis and the spermatic cord. The site of vasectomy was identified. The vas was held gently with Babcock forceps above and below the vasectomy site and a short segment was isolated, preserving the peri-vasal blood vessels. The distal end was divided and flushed with saline to confirm patency. The proximal end was then sharply divided, and the presence of sperms was confirmed under light microscopy. In the case of absence of sperms, a gentle barbotage of the proximal vas was performed and analyzed.

**Figure 1:**
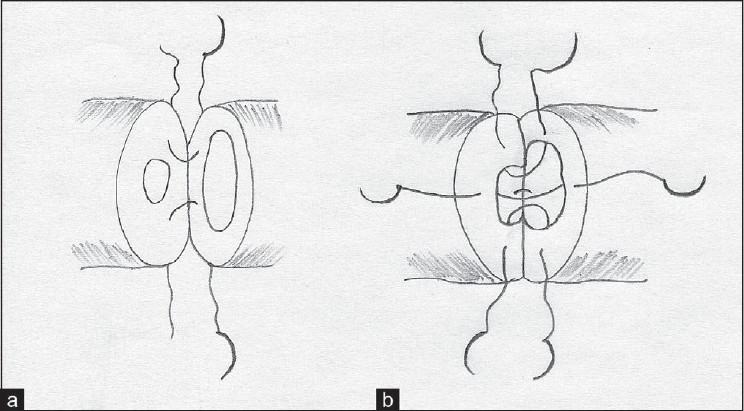
(a) Initial two outer-layer sutures. (b) Inner layer double-armed sutures: the 6 o’clock suture is pretied while the other three are tied together

Vasovasostomy was performed in two-layers. Two 8-0 polyamide sutures were placed at the 5 and 7 o’clock positions in the sero-muscular layer of both ends and tied [Figure 1a]. These two sutures also served to approximate the vasal ends for placement of the mucosal sutures. Next, a 10-0 double-armed polyamide suture was placed inside out through the mucosa, first on the proximal end and then on the distal end of the vas at the 6 o’clock position and tied. Three additional double-armed 10-0 polyamide sutures were sequentially placed at 3, 9, and 12 o’clock positions and tied once all three were in place [Figure 1b]. Subsequently two additional sero-muscular sutures were placed at 1 and 11 o’clock positions to complete the anastomosis. A third layer of supportive sutures was placed into the adventitia or peri-vasal tissue to enhance security of the anastomosis.

In those patients who had no fluid or sperms in the proximal end of the vas, the epididymis was exposed to identify the site of obstruction. These patients underwent a vasoepididymostomy using a previously described two-suture technique.[9]

Semen analysis was performed at 6 weeks after the surgery and three monthly thereafter till appearance of sperms in the ejaculate. Operative and recovery data were prospectively recorded into a database and reviewed for this report.

## RESULTS

During the period February 2008 to August 2009, eight men underwent vasectomy reversal using the 4 × 4 technique. Their demographic data are provided in Table 1. In six men, this technique was performed bilaterally with sperm seen in the proximal vas in all 12 anastomoses. In two men, sperms were present in the proximal vas on one side only. In this case, a vasovasostomy was performed only on one side. On the contralateral side, no sperm or fluid was present and they underwent a two-suture, longitudinal intussusception vasoepididymostomy on this side. Sperms were present in the epididymal fluid in both patients

**Table 1:** Patient data

Parameter	Value (range)
Age (years)	31 (21–38)
Duration of vasectomy (months)	40 (20–96)
Operative time (min)	90 (60–105)
Postoperative sperm density (mill/mL)	37.75 (15–70)
Postoperative motility (% Progressive)	39.37 (20–70)
Normal semen analysis (WHO criteria)	3

Mean operative time was 90 min per patient (range 60–105 min). There were no complications. All men had sperm in the ejaculate at the first semen analysis. Since routine follow-up was not performed after the initial semen analysis, pregnancy data are not available. Anecdotally, two patients from among the eight have confirmed pregnancies.

## DISCUSSION

The two basic principles of any luminal anastomosis are mucosa-to-mucosa approximation and a watertight closure. Approximation of the mucosal edges is important to allow healing while prevention of leakage is important, particularly for immunogenic substances, to minimize adhesions and scars. These principles are particularly relevant for a vasovasostomy since vasal fluid lacks the cellular elements of blood that could cause a clot formation and closure of the defect.[10]

Goldstein’s original meticulous technique using multiple sutures in three layers had some of the best reported results for this surgery.[7] However, the proposition of placing eight sutures in each of three layers in a tube where the diameter of the smaller one is 300 µm is a daunting task even for accomplished microsurgeons. This is probably the reason behind a large number of publications with differing techniques, each proposing to be easier than the other.

The two major areas of modification have been the use of a single-layer anastomosis and surgery with limited or no magnification. The single-layer procedures often do away with the need for microsutures and magnification. While Sharlip suggested a single-layer technique using 9-0 sutures carefully placed under a microscope, others have reported the one-layer technique without magnification and claimed excellent results.[4,11,12] However, one of the few experimental comparisons between the two-layer and single-layer techniques showed that the two-layer technique was superior not only in patency, but also in preventing damage to the testicular blood supply that might occur with the single-layer technique.[13] Some of the other attempts to simplify this procedure have looked at the option of using fibrin glue with only three sutures or a robotic-assisted procedure.[14–16] Clearly, there is no single technique which is superior to the other.

We believe that the best way to serve the dual purpose of adhering to the anastomotic principles and simplifying the procedure would be to follow a two-layered technique which is easy to perform. The use of magnification is necessary not only for placing precise, fine sutures for the vasovasostomy, but also to be prepared for the need for a vasoepididymostomy in a significant number of patients. Our current technique tends to serve both these objectives. The four inner sutures help obtain mucosal approximation whereas the outer four at staggered intervals cover the gaps in the inner layer and help make it watertight. Our use of eight sutures exactly matches the average number of sutures used by a group of 367 surgeons who reported routinely performing vasectomy reversals in a survey in 2004.[17] This survey also confirmed that the majority of surgeons prefer performing a two-layer technique, using a microscope and proceeding to a vasoepididymostomy in certain cases.

Our study is limited by the lack of pregnancy and follow-up data. We have alluded to this problem in our earlier reports on microsurgery for male infertility.[8,18] Three of our patients had normal seminal parameters at the first analysis whereas another two had marginal subnormal motility with normal sperm density. Interestingly, the two men with pregnant partners had neither normal counts nor normal motility at the first analysis.

## CONCLUSIONS

The “4 × 4” modified two-layer vasovasostomy is a simple technique that can be performed in quick time with excellent results. It adheres to the basic principles of luminal anastomosis and bridges the gap between a single- and two-layered procedures.
